# Temporary Loading Prevents Cancer Progression and Immune Organ Atrophy Induced by Hind-Limb Unloading in Mice

**DOI:** 10.3390/ijms19123959

**Published:** 2018-12-09

**Authors:** Akihisa Takahashi, Shoto Wakihata, Liqiu Ma, Takuya Adachi, Hiroki Hirose, Yukari Yoshida, Yoshinobu Ohira

**Affiliations:** 1 Gunma University Heavy Ion Medical Center, 3-39-22 Showa-machi, Maebashi, Gunma 371-8511, Japan; m1710006@gunma-u.ac.jp (S.W.); maliqiu@gunma-u.ac.jp (L.M.); skywatcher.tak@gmail.com (T.A.); m1810006@gunma-u.ac.jp (H.H.); yyukari@gunma-u.ac.jp (Y.Y.); 2 Faculty and Graduate School of Health and Sports Science, Doshisha University, 1-3 Tatara Miyakodani, Kyotanabe, Kyoto 610-0394, Japan; oyoshinobu@gmail.com

**Keywords:** tumor growth, metastasis, immune system, hind-limb unloading, animal model

## Abstract

Although the body’s immune system is altered during spaceflight, the effects of microgravity (μ*G*) on tumor growth and carcinogenesis are, as yet, unknown. To assess tumor proliferation and its effects on the immune system, we used a hind-limb unloading (HU) murine model to simulate μ*G* during spaceflight. HU mice demonstrated significantly increased tumor growth, metastasis to the lung, and greater splenic and thymic atrophy compared with mice in constant orthostatic suspension and standard housing controls. In addition, mice undergoing temporary loading during HU (2 h per day) demonstrated no difference in cancer progression and immune organ atrophy compared with controls. Our findings suggest that temporary loading can prevent cancer progression and immune organ atrophy induced by HU. Further space experiment studies are warranted to elucidate the precise effects of μ*G* on systemic immunity and cancer progression.

## 1. Introduction

To date, over 500 astronauts have traveled to space, with long-term stays of 6 months to 1 year in the International Space Station (ISS) likely to become possible. In the near future, manned space missions are scheduled to reach beyond low Earth orbit, such as return expeditions to the Moon, or to Mars. A mission to Mars will require spending approximately 2.5 years in space—6 months to travel there, 1.5 years on the surface, and 6 months to return. Space travel is no longer merely a dream. For safe long-term stays in space, it is urgent that we evaluate any detrimental effects on human physiological, behavioral, and psychological health to ensure astronaut health and performance under outer space-specific conditions.

Space radiation, including heavy ions, is one of the main health hazards of spaceflight. Exposure to space radiation on long-duration and exploration spaceflights may lead to an increased risk of cancer [1,2], tissue degeneration, and development of cataracts [3,4], and may affect the central nervous system [5,6,7], cardiovascular system [8], and immune functions [9]. Several factors, including microgravity (μ*G*) [10], are large uncertainties in the projection of these risks and prevent the evaluation of the effectiveness of possible countermeasures. Exposure to μ*G* were found to reduce bone [11], muscle [12], and ventricular masses [13], and “immune problems” were also associated with spaceflight [14,15,16,17,18,19]. In space shuttle experiments, spleen and thymic masses were reduced in flight mice [20], and significant changes in thymopoiesis was reported in healthy flight astronauts in association with a defined physiological, emotional, and physical stress event [21]. Immune system dysregulation has now been demonstrated to occur during spaceflight and persist during 6 months orbital spaceflights [17,22,23,24]. These results suggest that immune system aberrations caused by stressors associated with space travel should be included when estimating risk for pathologies such as cancer.

Hind-limb unloading (HU) of rodents was developed in the 1980s to enable the study of mechanisms, responses, and treatments for the adverse consequences of spaceflight. Although it is used to investigate the effect of weightlessness on the musculoskeletal system, several studies have suggested that HU has a similar impact on other physiological functions, including the immune system, to that experienced during anti-orthostasis and inactivity [25,26,27,28]. Although immunodeficient mice showed no difference in tumor growth, normal mice demonstrated significantly increased tumor growth and greater splenic atrophy during HU compared with controls [29].

In this study, we assessed metastasis in HU mice to investigate cancer progression under μ*G*. In addition, we verified how to prevent cancer progression during HU.

## 2. Results

### 2.1. Change of Body Weight by Four Suspension Conditions

Mice in the suspension groups (HU, temporary loading during HU (TL), and orthostatic suspension (OS)), demonstrated reduced body weight compared with the standard housing group (Con). At 3 days after suspension, there were no statistically significant differences in body weight between the suspension groups. In addition, there were no significant differences in body weight between the HU and TL groups at 21 days after the inoculation of cancer cells (Table 1).

### 2.2. Temporary Loading Prevents Immune Organ Atrophy by Hind-Limb Unloading

**Figure 1:**
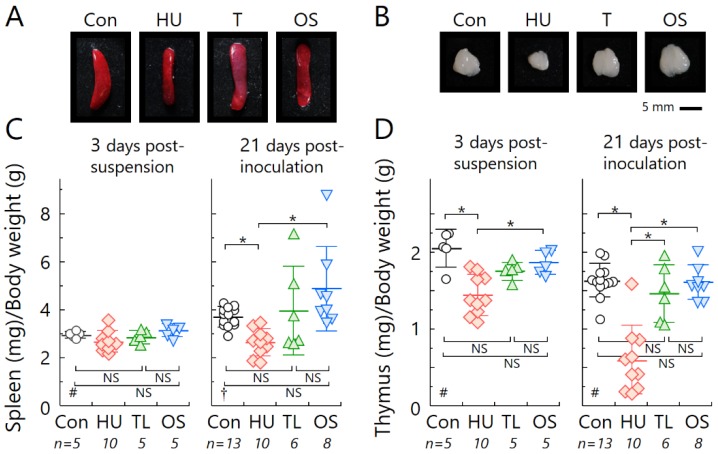
Lymphoid organ atrophy in the four suspension conditions was measured at 3 days after suspension and at 21 days after tumor cell inoculation. (**A**,**B**) Representative photographs at 21 days after tumor cell inoculation. (**C**,**D**) Organ weight relative to body weight. (**A**,**C**) Spleen. (**B**,**D**) Thymus. Circles, standard housing control group (Con); diamonds, hind-limb unloading (HU); triangles, temporary loading during HU (2 h per day) (TL); inverted triangles, orthostatic suspension (OS). Error bars indicate standard errors. ^#^, ANOVA test; ^†^, Kruskal-Wallis test. * *p* < 0.05; NS, not significant.

**Figure A1:**
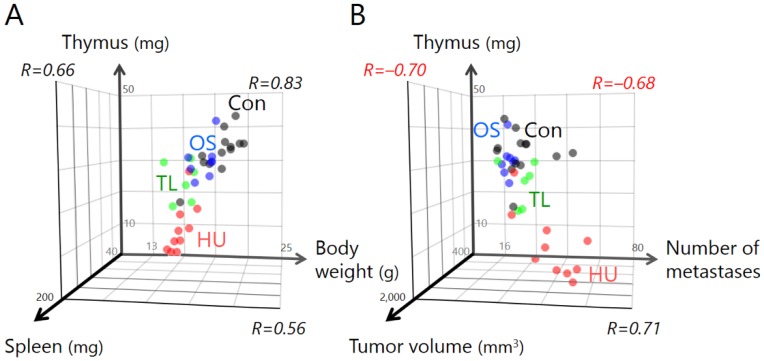
Scatterplot and correlation in four suspension conditions at 21 days after inoculation of LM8 cells. (**A**) Positive correlation between body weight, spleen weight, and thymus weight; (**B**) negative correlation between number of metastases or tumor volumes, and thymus weight. Black dots, standard housing control (Con) (*n* = 13); red dots, hind-limb unloading (HU) (*n* = 10); green dots, temporary loading during HU (2 h per day) (TL) (*n* = 6); blue dots, orthostatic suspension (OS) (*n* = 8). R, correlation coefficient.

The spleen and thymus in HU mice were shrunken compared with the other experimental groups (Figure 1). Because it was thought that weight loss influenced the size of these organs, we calculated the fresh weight of the spleen and thymus relative to body weight. The weights of these organs in HU mice were significantly lower than those of the Con and OS groups, although there was a positive correlation between body weight and organ weight (Figure A1A). However, no significant differences in splenic or thymic mass were seen between TL mice and Con or OS groups (Figure 1).

### 2.3. Temporary Loading Prevents Acceleration of Tumor Growth by Hind-Limb Unloading

**Figure 2:**
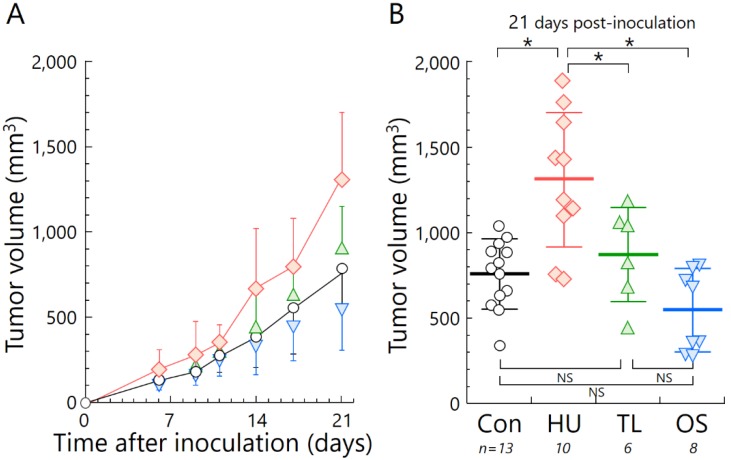
Tumor growth of mice in the four suspension conditions. (**A**) Tumor volume after tumor cell inoculation and (**B**) at day 21 post-inoculation. Circles, standard housing control group (Con); diamonds, hind-limb unloading (HU); triangles, temporary loading during HU (2 h per day) (TL); inverted triangles, orthostatic suspension (OS). Error bars indicate standard errors. * *p* < 0.05; NS, not significant (using ANOVA test).

Tumor growth in the HU group was significantly accelerated compared with that of the other experimental groups. TL mice had slower tumor growth compared with HU mice. In addition, there were no statistically significant differences in tumor growth between the TL and Con or OS groups (Figure 2).

### 2.4. Temporary Loading Prevents Acceleration of Metastasis by Hind-Limb Unloading

**Figure 3:**
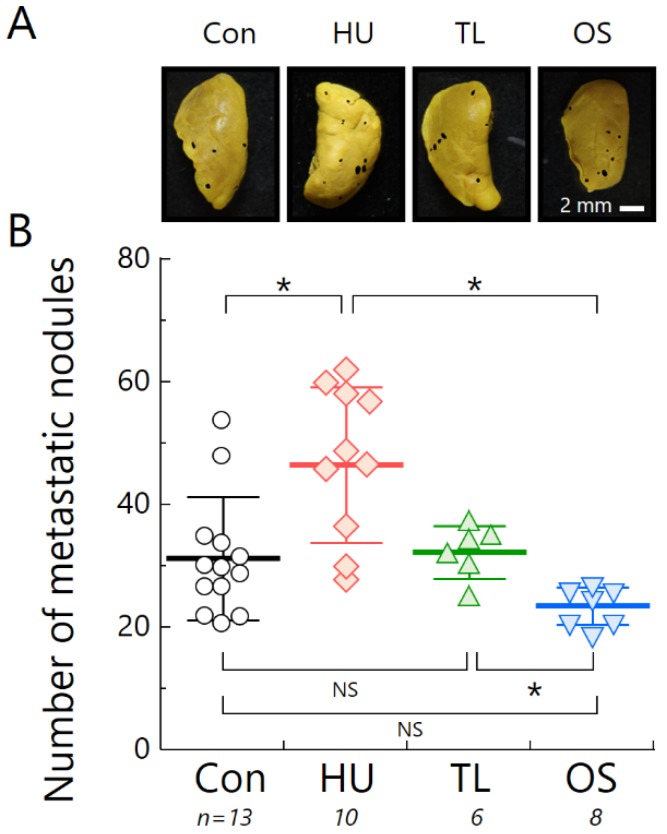
Number of lung metastatic nodules found under the four suspension conditions at 21 days after inoculation with murine osteosarcoma cell line (LM8) cells. (**A**) Representative photographs. Lung metastases are delineated and shaded in black. (**B**) Number of metastatic nodules. Circles, standard housing control group (Con); diamonds, hind-limb unloading (HU); triangles, temporary loading during HU (2 h per day) (TL); inverted triangles, orthostatic suspension (OS). Error bars indicate standard errors. * *p* < 0.05; NS, not significant (using Kruskal-Wallis test).

The number of metastatic nodules was higher in HU mice compared with that of the other experimental groups. The TL group demonstrated 32.1% fewer metastatic nodules compared with HU, and there were no statistically significant differences in the number of metastases between the TL and Con groups. Although there were no statistically significant differences between the Con and OS groups, the number of metastases in the OS group was significantly lower than the other suspension groups (Figure 3). Additionally, a negative correlation between immune organ weight and cancer progression was also identified (Figure A1B).

## 3. Discussion

In this study, we demonstrated the effects of HU on immune organ atrophy (Figure 1) and the accelerated tumor growth of osteosarcoma in vivo (Figure 2). Our data agree with a previous report using spindle cell carcinoma in the HU mouse model [29]. To clarify the potential for metastasis under HU, we used LM8 cells with high metastatic potential to the lung [30]. Increased lung metastasis during HU in our experiment can almost certainly be explained by changes in anti-tumor immune responses (Figure 3). There was also a negative correlation between immune organ weight and indicators of cancer progression, such as tumor volume and number of metastases (Figure A1B). Immune organ atrophy may be caused by hormones such as sclerostin and osteopontin through the loss of mechanical loading to the bones [26,31]. It was reported that the multifunctional hormone osteopontin plays diverse roles in bone biology, immune regulation, and cancer metastasis [26]. Many studies have investigated virus infection in relation to immune system dysregulation during spaceflight or HU [32,33,34], but there is currently very little data regarding cancer progression [35]. The immune system usually protects the body from tumor initiation to metastatic progression by the destruction of abnormal cells [36]. The current study suggests the possibility that prolonged μ*G* of a long-term stay in space may increase the risk of cancer incidence and mortality.

Space radiation is a cause of increased cancer risk [1,2]. During a long-term deep space mission outside Earth’s protective magnetic field, astronauts will be constantly exposed to galactic cosmic rays (GCRs) and occasionally to particles from large solar particle events. Because the energy of some GCR particles is very high, it is difficult to protect astronauts using conventional materials [37]. This phenomenon may increase the risk of cancer development in conjunction with extended μ*G* duration. Importantly, cancer risk assessment for space radiation based on the dose response data of static radiation conditions with disregard to the influence of μ*G* might underestimate the potential risk posed to astronauts. In the near future, astronauts and civilians who might harbor undetectable micro-cancers may undertake long-term stays in space. Therefore, such increased cancer risk poses a significant problem.

This finding raises another unresolved question: How can we prevent cancer progression induced by μ*G*? To answer this, we investigated the effect of TL on lymphoid organ atrophy and cancer progression. We found significant differences between the TL and HU groups using the Student’s *t*-test. This new finding indicates that TL prevents the negative effects of μ*G*. Interestingly, astronauts routinely undertake physical exercise for an average of 2 h per day, incorporating both strength and aerobic training to counteract reductions in muscle strength, mass, and cardiorespiratory fitness that occur because of prolonged periods in μ*G* spaceflight (Figure 1, Figure 2 and Figure 3). It was reported that an additional benefit of performing exercises in space is that it has profound effects on the normal function of the immune system [19,38]. Indeed, exercise was shown to increase the release of certain “myokines”, such as IL-7, which is essential for maintaining thymic function and stimulating the release of new T-cells [39].

Our research HU methods have a significant limitation; HU may not represent a perfect model of μ*G*. Therefore, it will be necessary to verify these results in space-based experiments after feasibility studies have been performed. The space experimental environment was well-regulated using newly developed mouse habitat cage units, which were installed in the Multiple Artificial-gravity Research System on the ISS, and enabled mice to be exposed to μ*G*, partial gravity, and 1*G* conditions [11]. These space experiments are critically important to clarify the possibility of cancer progression induced by immune system dysregulation, and to increase our knowledge and promote technological advances to counteract human adaptation during and after prolonged deep spaceflight.

## 4. Materials and Methods

### 4.1. Mice

Female C3H/HeNJcl mice (7 weeks old) were obtained from Clea Japan, Inc. (Tokyo, Japan). Mice were housed in individual cages in a temperature- and humidity-controlled (23 ± 1 °C and 60 ± 5% relative humidity) room with a 12 h (6 am–6 pm) light–dark cycle. All experimental animals were procured, maintained, and used in accordance with the Recommendations for Handling of Laboratory Animals for Biomedical Research, compiled by the guidelines of the Animal Care and Experimentation Committee of Gunma University, Showa Campus (No. 18-023; Application date: 19 March 2018).

### 4.2. Cell Culture

A murine osteosarcoma cell line (LM8) was obtained from the RIKEN BioResource Research Center (Tsukuba, Japan). The cells were cultured in Dulbecco’s modified Eagle’s medium containing high glucose and L-glutamine, and supplemented with 10% (*v/v*) heat-inactivated fetal bovine serum (FBS), penicillin (100 U/mL), streptomycin (100 μg/mL), and 4-(2-hydroxyethyl)-1-piperazine ethanesulfonic acid (10 mM) at 37 °C in a humidified atmosphere of 5% CO_2_.

### 4.3. Tail Suspension

**Figure 4:**
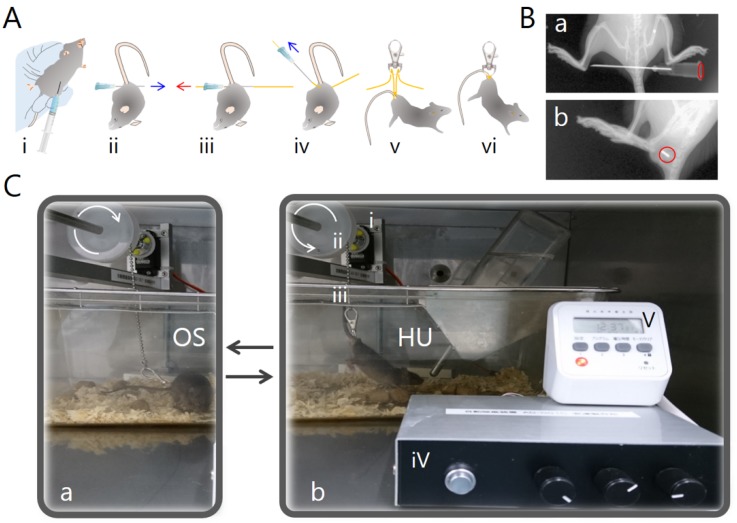
Tail suspension. (**A**) Method of tail suspension. (**i**) Mice were anesthetized by intraperitoneal injection of 37 mg/kg ketamine and 7.4 mg/kg xylazine in 250 μL saline. (**ii**) A needle was placed into the sacrum coccyx joint of the mouse, (**iii**) threaded, and (**iv**) removed, leaving the thread in place. (**v**) The nylon thread was fed through a rotary hook, and (**vi**) tied in place to suspend the hind limbs of the animal. (**B**) Transmission image at needle puncture site using a Kodak IS4000 IN-VIVO FX (Carestream Health Inv., Rochester, NY). (**a**) Top view; (**b**) view from the side. Red circle, needle base. (**C**) An electric suspension device with a digital power supply timer. (**a**) Orthostatic suspension (OS). (**b**) Hind-limb unloading (HU). (**i**) motor; (**ii**) fixed pulley; (**iii**) rope; (**iv**) controller; and (**v**) digital power supply timer.

Tail suspension is the most commonly used animal model of μ*G* in outer space. Prior to tail suspension experiments, mice were allowed to acclimatize to being housed individually in single cages (width 200 × depth 300 × height 130 mm) before suspension. Briefly, a small (35 × 13 mm) metallic rotary hook (PandaHall, Guangdong, China) was linked together with a nylon thread of 0.29 mm diameter (CN500, DUEL Co., Inc., Fukuoka, Japan) by puncturing a 23G needle (Terumo Corp., Tokyo, Japan) into the sacrum coccyx joint of the mouse (Figure 4A,B).

The hook was then attached to a small swivel key chain that was connected to an electric suspension device with a digital power supply timer (AD-001, Adachi Factory, Maebashi, Japan). Mice could move on the y-axis and rotate 360 degrees, and therefore had access to all areas of the cage. Tail suspension is widely performed by placing adhesive tape around the tail [40,41]; however, necrosis of the tail end often occurs if the blood flow is inhibited by the tape. Therefore, we used the hook and key chain method because such detrimental effects were not observed in response to this type of hind-limb suspension (Ohira et al., unpublished data). Mouse hind-limbs were maintained just off the cage floor with the body of the mouse at an angle of approximately 30° from the cage floor. The mice could move freely, and the angle and height of the mice were checked daily (HU group, Figure 4C(b)). The TL mice were released from suspension for 2 h (8 pm–10 pm) per day using an electric suspension device digital power supply timer (TL group).

The orthostatic suspension mice were separated into individual cages under identical conditions to the unloading groups, but without tail suspension (OS group, Figure 4C(a)). As a control experiment, mice were kept under standard housing conditions without introduction of a thread into the tail (Con group).

### 4.4. Experimental Schedule

**Figure 5:**
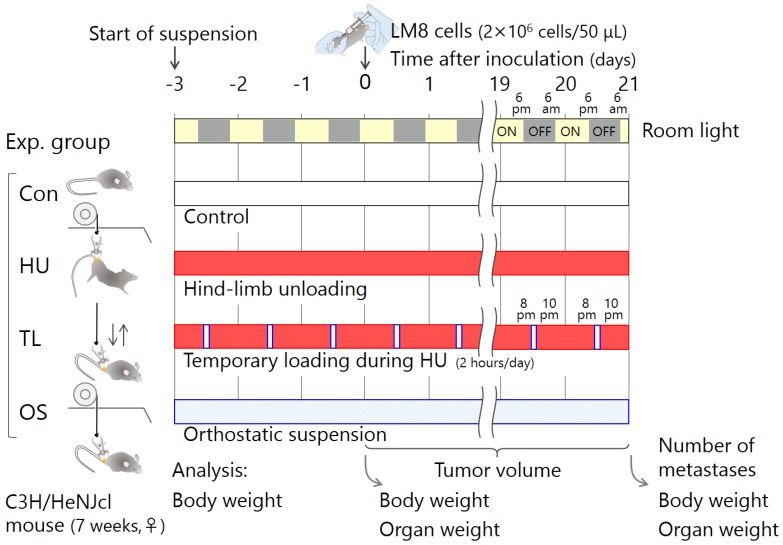
Schematic work flow for experiments. LM8 cells, a murine osteosarcoma line, were inoculated subcutaneously in C3H/HeNJcl mice 3 days after tail suspension under four conditions: Con, standard housing control; HU, hind-limb unloading; TL, temporary loading during HU (2 h per day); and OS, orthostatic suspension.

A schematic of the work flow for experiments is shown in Figure 5. The mice were divided into four groups—Con, HU, TL, and OS. LM8 cells (2 × 10^6^ cells in 50 μL culture medium without FBS, administered by subcutaneous injection) were inoculated into the lower right abdominal region on day 3 after tail suspension. We measured tumor size regularly throughout the experiment, and quantified the number of lung metastatic nodules at 21 days post-inoculation. To assess whether the spleen and thymus were diminished at 3 days after tail suspension (*n* = total 25) and at 21 days after LM8 inoculation (*n* = total 37), the spleens and thymi were weighed.

### 4.5. Measurement of Tumor Growth

The diameters of tumors (length and width) were measured using a vernier caliper at the time of treatment, and twice a week thereafter. The lengths and widths obtained by superficial two-dimensional measurements were recorded. Tumor volume (TV) in mg was calculated according to the formula TV = (4/3) × π × L × W^2^, where L and W are the length and width in mm, respectively.

### 4.6. Measurement of Lung Metastatic Nodules

Bilateral lungs were initially fixed in Bouin solution overnight at day 21 after subcutaneous tumor cell implantation into the mice. Pulmonary metastatic nodules on the surfaces of all the pulmonary lobes were macroscopically counted.

### 4.7. Statistical Analysis

All values were expressed as the mean ± standard deviation (SD), with n indicating the number of independent experiments. EZR (Easy R) free software (version 1.37) was used for statistical analysis [42]. The Bartlett test was used to analyze the normal distribution of data. Differences in demographic data among the groups were analyzed using one-way analysis of variance (ANOVA) or the Kruskal-Wallis test (non-parametric equivalent of the ANOVA) for continuous variables, in accordance with the data normality. To analyze differences in knowledge, attitudes, and behaviors among the different occupational categories, Tukey’s test was used for the post-hoc analysis of parametric variables analyzed using ANOVA, and post-hoc comparisons for non-parametric variables analyzed using the Kruskal-Wallis tests were made using the Steel-Dwass multiple comparison test. A *p* value of less than 0.05 was considered statistically significant.

## 5. Conclusions

Our study demonstrates the induction of cancer progression and lymphoid organ atrophy by HU. Of note, temporary loading prevented these adverse effects. This finding may have important implications for long-term space travel. It is necessary to verify these findings by performing experiments in space.

## Figures and Tables

**Table 1:**
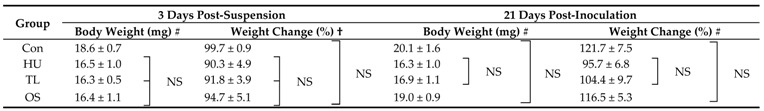
Body weight change in four suspension conditions measured at 3 days after suspension and 21 days after tumor cell inoculation.

Con, standard housing control; HU, hind-limb unloading; TL, temporary loading during HU (2 h per day); OS, orthostatic suspension. Weight changes (% ± standard error) were calculated using the body weight before and after treatment. ^#^, ANOVA test; ^†^, Kruskal-Wallis test. NS, not significant.

## Abbreviations

Con	No suspension control
FBS	Fetal bovine serum
GCR	Galactic cosmic rays
GHMC	Gunma University Heavy Ion Medical Center
HU	Hind-limb unloading
ISS	International Space Station
OS	Orthostatic suspension
TL	Temporary loading during hind-limb unloading
μ*G*	Microgravity
